# Visualisation of cerebrospinal fluid flow patterns in albino *Xenopus *larvae *in vivo*

**DOI:** 10.1186/2045-8118-9-9

**Published:** 2012-04-25

**Authors:** Kazue Mogi, Takeshi Adachi, Susumu Izumi, Ryuji Toyoizumi

**Affiliations:** 1 Research Institute for Integrated Science, Kanagawa University, Tsuchiya 2946, Hiratsuka city 259-1293, Japan; 2 Department of Biological Science, Kanagawa University, Tsuchiya 2946, Hiratsuka city 259-1293, Japan

**Keywords:** CSF flow, Dorso-ventral asymmetry, Left-right asymmetry, Brain ventricle, *Xenopus laevis*, Albino larva, Visualisation, Qdot nanocrystals, Polystyrene beads

## Abstract

**Background:**

It has long been known that cerebrospinal fluid (CSF), its composition and flow, play an important part in normal brain development, and ependymal cell ciliary beating as a possible driver of CSF flow has previously been studied in mammalian fetuses *in vitro*. Lower vertebrate animals are potential models for analysis of CSF flow during development because they are oviparous. Albino *Xenopus laevis *larvae are nearly transparent and have a straight, translucent brain that facilitates the observation of fluid flow within the ventricles. The aim of these experiments was to study CSF flow and circulation *in vivo *in the developing brain of living embryos, larvae and tadpoles of *Xenopus laevis *using a microinjection technique.

**Methods:**

The development of *Xenopus *larval brain ventricles and the patterns of CSF flow were visualised after injection of quantum dot nanocrystals and polystyrene beads (3.1 or 5.8 μm in diameter) into the fourth cerebral ventricle at embryonic/larval stages 30-53.

**Results:**

The fluorescent nanocrystals showed the normal development of the cerebral ventricles from embryonic/larval stages 38 to 53. The polystyrene beads injected into stage 47-49 larvae revealed three CSF flow patterns, left-handed, right-handed and non-biased, in movement of the beads into the third ventricle from the cerebral aqueduct (aqueduct of Sylvius). In the lateral ventricles, anterior to the third ventricle, CSF flow moved anteriorly along the outer wall of the ventricle to the inner wall and then posteriorly, creating a semicircle. In the cerebral aqueduct, connecting the third and fourth cerebral ventricles, CSF flow moved rostrally in the dorsal region and caudally in the ventral region. Also in the fourth ventricle, clear dorso-ventral differences in fluid flow pattern were observed.

**Conclusions:**

This is the first visualisation of the orchestrated CSF flow pattern in developing vertebrates using a live animal imaging approach. CSF flow in *Xenopus *albino larvae showed a largely consistent pattern, with the exception of individual differences in left-right asymmetrical flow in the third ventricle.

## Background

Early investigators studied the role of cerebrospinal fluid (CSF) flow and observed that CSF flow is generated by a balance of fluid dynamics between the CSF pressure and CSF absorption by tissues [1]. More recently, the use of magnetic resonance imaging (MRI) revealed that ciliary movement induces a constant CSF flow in humans [2-4]. Previous investigators also reported the ciliary action of ependymal cells in amphibians [5]. Abnormal CSF flow causes various diseases in mammals, such as communicating hydrocephalus in humans where the CSF flow differs from that of healthy individuals [6-9]. These observations suggest that continuous flow may be necessary for the normal development and maintenance of brain function.

In lower vertebrates, the ventricle structures and the existence of CSF flow have been investigated using morphological and/or physiological approaches. It has been shown by scanning and/or transmission electron microscopy that a fenestrated ependymal cell layer exists in the caudal end of the roof of the fourth cerebral ventricle in four amphibian species [10] and in embryos and larvae of *Rana pipiens *[11]. Furthermore, CSF flows between the fenestrated ependyma of the fourth ventricle and subarachnoid space [12,13]. However, detailed studies of the pattern and dynamics of CSF flow using live imaging of embryonic, larval and/or adult amphibian brains have not been reported. Thus, little is known about the biological significance of the CSF flow pattern in lower vertebrates, in general, or about the role of CSF flow in early neurogenesis and for the establishment of ependymal cells' planar cell polarity [14-16]. In this report, albino larvae of *Xenopus laevis *are shown to be a good model organism for monitoring the CSF flow pattern using a simple microinjection approach. *Xenopus laevis *albino larvae have transparent skin, a translucent brain and large cerebral ventricles that facilitate observation. Using albino individuals of *Xenopus laevis*, the visualisation of CSF flow in a living animal has the potential to generate novel findings elucidating the role of early CSF circulation during brain development and neurogenesis. The aim of these experiments was to study CSF flow and circulation *in vivo *in the developing brain of living embryos, larvae and tadpoles of *Xenopus laevis *using a microinjection technique. This is the first report showing left-right and dorso-ventral asymmetries in CSF flow within a ventricle, and the results demonstrate that the *Xenopus *albino larvae are a model organism suitable for analysing the biological role of the CSF flow pattern. Our approach provides an opportunity for CSF investigators to examine the relationship between physiological flow patterns and growth/maturation of the central nervous system.

## Methods

Fertilised eggs of albino *X. laevis *were incubated in 10% Steinberg's solution (58 mM NaCl, 0.67 mM KCl, 0.44 mM Ca(NO_3_)_2_, l.3 mM MgSO_4_, 4.6 mM Tris-HCl, pH 7.4) before reaching the larval stages [17]. Larvae were reared with vegetable juice in pond water at 26°C. Larvae were anesthetised in 10% Steinberg's solution containing 0.01% MS-222 (ethyl 3-aminobenzoate methanesulfonate, CAS number 886-86-2), before injections. After the injection, they were photographed and then transferred into normal 10% Steinberg's solution where they woke up spontaneously from the anesthetic and started to swim normally within 1 h.

### Visualisation of *Xenopus *larval brain ventricles using nanocrystals

Using a Nanoject instrument (Drummond Scientific, Broomall, PA, USA), Qdot565 solution containing 2 μM quantum dot nanocrystals **(**Qtracker^®^565 non-targeted quantum dots 2 μM solution, catalog Q21031MP, Quantum Dot Co., Hayward, CA, USA) was injected. In the stock solution, Qdot565 was suspended in 50 mM borate buffer (pH 8.3), and the stock solution was injected without dilution. Injection volumes were 4.6 or 9.2 nL per embryo at stage 30 to 38, and 23 or 46 nL per larva at later developmental stages (stage 41-51), or for tadpoles with well-developed hind limbs (stage 53). Numbers injected were: stage 30-38, n = 9; stage 41-43, n = 19; stage 46-48, n = 19; stage 50-51, n = 12; stage 53, n = 8. The Nanoject specifications enable the injection of volumes with a precision of two significant digits. The injected larvae were observed using a fluorescence stereoscopic microscope (Olympus, SZX12, Tokyo, Japan), using excitation at 460 to 490 nm, and photographed with a digital camera (Olympus, DP-70).

### Visualisation of the CSF flow pattern in the larval *Xenopus *brain using polystyrene beads

**Figure 1:**
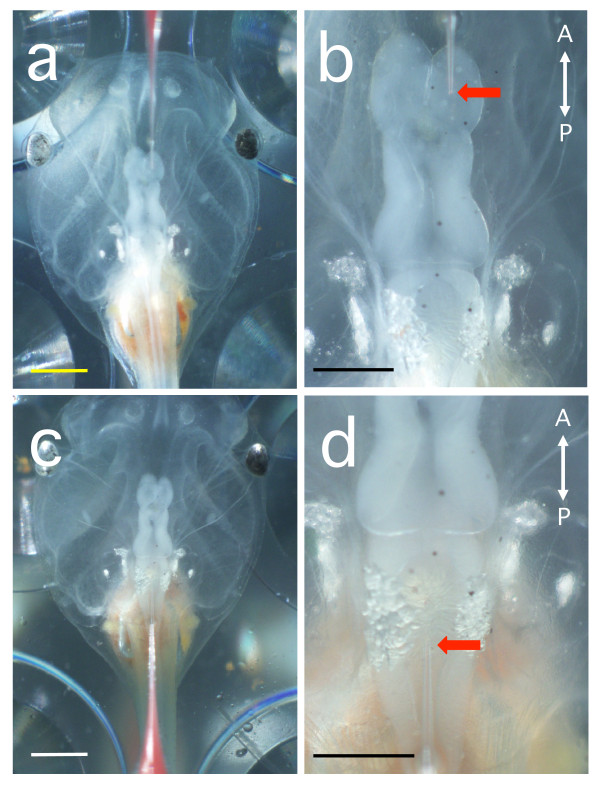
**Sites for the injection of polystyrene beads into the ventricles of anesthetised *Xenopus *larvae**. A glass capillary filled with an aqueous suspension of red beads (2.9 μm in diameter) was inserted into the lateral ventricle **(a, b) **or the fourth ventricle **(c, d) **for injections. Arrows indicate the injection points. **(b, d) **magnified views of **(a, c)**, respectively. Scale bars, 1 mm (a, c) and 0.5 mm (b, d).

An aqueous solution containing polystyrene beads was injected into the fourth ventricle of albino *Xenopus *larvae at stages 47-49 (Figure 1, n = 479). Two kinds of white beads and blue beads were mainly used for the analysis of CSF flow pattern in the larval brain (white beads, Polybead^® ^Microspheres 3.00 μm, Polysciences Inc., catalog # 17134, Warrington, PA, USA; blue beads, Polybead^® ^Polystyrene Blue Dyed Microsphere 6.00 μm, Polysciences Inc., catalog # 15715) and the bead stock solution was suspended in water at a bead concentration of 2.5%. The average diameter of the beads was as follows; white: 3.135 ± 0.146 μm, blue: 5.801 ± 0.417 μm, mean ± SD, c. v. was less than 10%, determined to be optimal for observation of CSF flow. Since the beads often aggregate and rapidly clog the injection capillary, they were pretreated with 1% bovine serum albumin (BSA). Suspended beads (4 μL) and 1% BSA solution (100 μL) were mixed and vortexed for 1 min, then centrifuged for 30 sec and the supernatant removed. The beads were rinsed 2-3 times in ultrapure water and were diluted in 8 μL of the ultrapure water. The solution was poured into the well of mini tray (Nunc, catalog 439225, Rochester, NY, USA) and aspirated into the paraffin-filled capillary for the injection. Nanoject can be used for quantifiable injection because one push of the injection button makes the correct advance of the stainless needle-like plunger within the paraffin-filled glass capillary for 2.3 nL or 4.6 nL injection volumes according to the DIP-switch position, and thus we could quantify the injection volume by the number of times the injection button was pressed. In this report, the beads were injected at a volume of 4.6 or 23 nL by serial injection of the unit volumes (2.3 nL or 4.6 nL).

Using an Olympus SZX12 stereoscopic microscope with a CCD camera or, in some cases, an Olympus IX71 inverted microscope with relief contrast objectives, the movement of the injected beads was observed in real-time and recorded with a VHS video recorder (Panasonic, Osaka, Japan) or a DP21 CCD camera controller (Olympus). Image-J software http://rsbweb.nih.gov/ij/download.html and its plug-in "Color Footprint Rainbow" http://www.jaist.ac.jp/ms/labs/hiratsuka/index.php were used to visualise bead movements as multi-coloured video-tracking images.

### Check assays for injection conditions and examination of stage-dependency

To determine whether the injection of beads into the fourth ventricle affected the endogenous CSF flow pattern, we injected beads into the lateral cerebral ventricle. In preliminary experiments, we determined the appropriate colour and particle size of the beads injected into embryos or young larvae before stage 47. Then, it was examined whether stage-dependent change of CSF flow pattern occurs using stage 38-39, stage 42-43, stage 46 embryos/young larvae, and stage 47-49 larvae.

We checked whether or not the injection of beads *per se *affected endogenous CSF flow patterns. Beads with different diameters were injected at two separate times to determine whether the first injected beads changed the flow pattern of the beads in the second injection. Blue beads (diameter = 5.8 μm) were injected into the fourth cerebral ventricle, and after 30 min, red beads (diameter = 2.9 μm) were injected into the same site, and CSF flow was observed. The beads were differentiated by colour and particle size during observations, and flow patterns compared after the first and second injections.

### Estimation of the volume of the larval brain ventricle

To estimate the volume of the cerebral ventricles, sesame oil was injected into the third or fourth ventricle. Sesame oil is yellow in colour, remains in one large droplet and has minimal toxicity. A pinhole was made in the fourth cerebral ventricle using a glass capillary before injection of the sesame oil into the third ventricle. Conversely, a pinhole was made in the third ventricle, and oil was then injected into the fourth cerebral ventricle. When oil was injected into the third ventricle of stage 46 larvae without making a pinhole in the fourth ventricle before the oil injection, the oil could not penetrate into the caudal tip of the fourth cerebral ventricle and *vice versa *when oil was injected into the fourth ventricle. Pinholes enabled the injected oil to completely fill the ventricles, including both lateral cerebral ventricles and the caudal tip of the fourth ventricle.

To estimate the volume of larval brain ventricle, the number of injections required to fill up completely the ventricular space without leakage was noted. For example, when *n *times of the 4.6 nL unit injection filled the ventricles without leaking, while *n + 1 *times of the same injection caused leakage of oil, then ventricular volume for the injected larva was estimated to be between 4.6X*n *nL and 4.6X(*n + 1*) nL.

## Results

### Visualisation of ventricular morphology using Qdot nanocrystals

**Figure 2:**
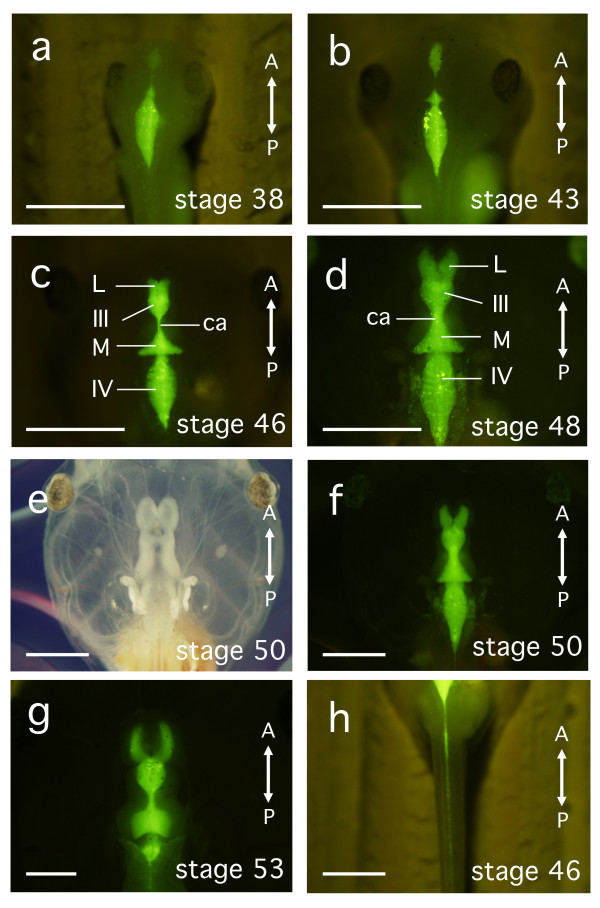
**Morphogenesis of the cerebral ventricles during *X. laevis *development at stages 38-53, visualised by Qdot565 nanocrystals**. The injection into the fourth cerebral ventricle of anesthetised embryos labelled the fourth cerebral ventricle almost exclusively in stage 38 **(a) **and stage 43 **(b) **embryos. The third cerebral ventricle (III) forms an oval shape by stage 43, and the midbrain ventricle (M) is visible between the fourth and third ventricles by stage 46. The lateral ventricles (L) bud off the third ventricle at stages 46 to 48 **(c, d)**. All ventricles can be recognised clearly at stage 50 (bright-field; **e**, fluorescence; **f**). The morphology of the ventricles in stage 53 larvae is different from those of early larval stages **(g)**. The central canal of the spinal cord is also labelled **(h)**. All panels are dorsal views with anterior at the top. Scale bars, 1 mm. ca: cerebral aqueduct.

Injecting Qdot565 nanocrystals into the fourth cerebral ventricle very clearly labelled the ventricles, which resulted in their easy visualisation (Figure 2). While the ventricular space seemed to be nearly closed in stage 30 to 34 embryos, it had opened slightly along the anteroposterior axis by stage 35 to 36. In stage 38 tailbud embryos, the fourth ventricle was labelled and by stage 43 there was also a small elliptical frontal extension of the third ventricle (Figure 2a). Frontal budding of the lateral ventricles was not yet apparent in stage 38 to 43 embryos (Figures 2a and 2b). The lateral ventricles became additionally visible at stage 46 as a left-right separation at the anterior of the third cerebral ventricle (Figure 2c). While the fourth cerebral ventricle was slightly oval in the younger stages, it became rhomboid in shape by late stage 48 (Figures 2d, e and 2f). In stage 53 larvae, which had initiated hindlimb and forelimb development, the lateral ventricles and the cerebral aqueduct narrowed, and the fourth ventricle lost transparency due to growth of the cranial cartilage (Figure 2g). For many of the larvae injected with Qdot565, the central canal of the spinal cord was also labelled (n = 9 out of 19 for stage 46-48, n = 16 out of 20 for stage 50-53; Figure 2h), indicating that the ventricular CSF flows into the spinal cord.

### Pattern of CSF flow in the larval ventricles and the cerebral aqueduct (stage 47-49)

**Figure 3:**
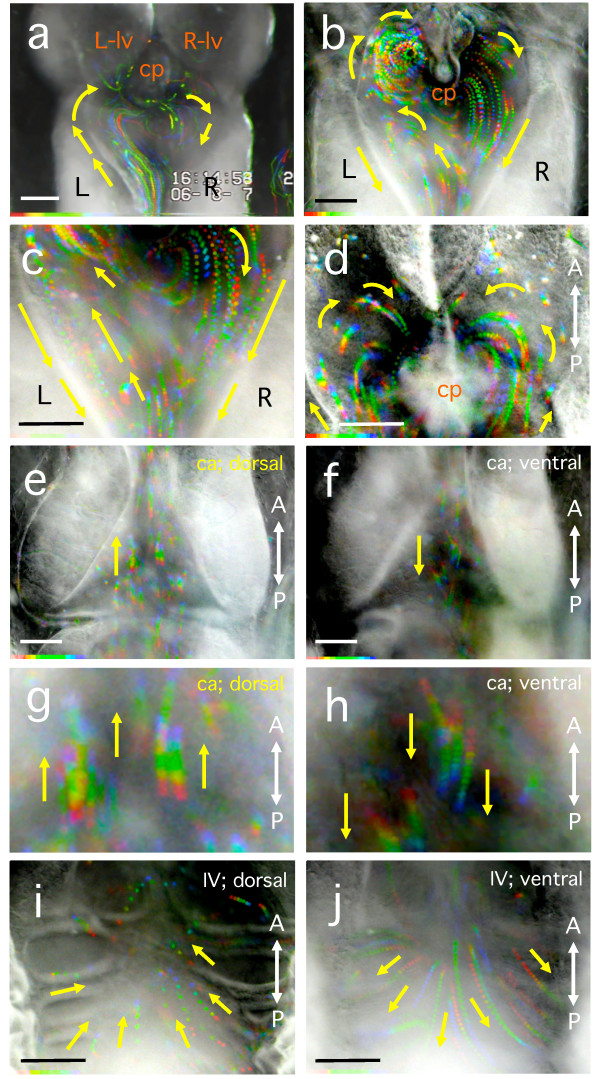
**Visualisation of fluid flow by injecting polystyrene beads into the cerebral ventricles of anaesthetised albino *X. laevis *larvae at stage 47-49**. Serial multi-coloured dots represent the trajectories of each bead from 0.7 to 5.3 seconds. The brightness of the video frames are inverted, and the trajectories of the beads were visualised by red - > orange - > yellow - > green - > blue - > purple, in turn, using a combination of Image-J software and its plug-in "Color Footprint Rainbow". **(a) **The beads diffused into the third cerebral ventricle mainly along the left wall of the ventricle just after the injection. **(b, c) **The beads moved with left-right asymmetrical circulation in the third ventricle (b) and exited through the cerebral aqueduct along the wall of the third ventricle (c). In many individuals, a left-right asymmetry in bead movements in the third ventricle was observed (see Table 1 and Table 2). **(d) **Within the lateral ventricles, the beads move along the distal walls toward the medial walls creating a semi-circular movement. **(e-h) **In the cerebral aqueduct, the beads moved rostrally in the dorsal region [(e) and (g) magnified view of (e)] and caudally in the ventral region [(f) and (h) magnified view of (f)]. **(i, j) **In the fourth ventricle [(i) dorsal side; (j) ventral side], beads tended to concentrate toward the centre and move to the anterior in the dorsal region, whereas beads tended to flow toward the sides and move to the posterior in the ventral region. cp: choroid plexus; lv: lateral ventricle; ca: cerebral aqueduct (aqueduct of Sylvius). All panels are dorsal views (see Additional file 1: movie 1, Additional file 2: movie 2, Additional file 4: movie 4, Additional file 5: movie 5, Additional file 6: movie 6, Additional file 7: movie 7, Additional file 8: movie 8, and Additional file 9: movie 9). Duration of recording; (a) 5.3 sec; (b) 1.0 sec; (c) 0.9 sec; (d) 1.2 sec; (e) 0.9 sec; (f) 0.7 sec; (i) 1.5 sec; (j) 2.6 sec. Scale bars, 0.1 mm.

CSF flow in the cerebral ventricles of stage 47-49 larvae was visualised by injecting larvae with a suspension of BSA-coated polystyrene beads. The pattern of CSF flow was observed from the dorsal side through the translucent brain. Within 30 s after injection into the fourth ventricle, the beads passed through the cerebral aqueduct (Figure 3a, Table 1, Additional file 1: movie 1), and moved into the third cerebral ventricle as a result of CSF circulation (Figure 3b, Table 2, Additional file 2: movie 2). When the beads first entered the third cerebral ventricle from the cerebral aqueduct, they tended to either move along either the left wall or the right wall of the ventricle (left-handed, 41%, n = 190/467; right-handed, 20%, n = 95/467, Table 1, Additional file 1: movie 1). In the remaining cases, no-asymmetrical movement was observed and bead movement was either random or straight into the centre of the ventricle (Table 1). In most of the observed larvae, the CSF flow around the choroid plexus was faster than in other regions of the third cerebral ventricle, suggesting that ciliary movement in the choroid plexus is more active than in the other regions. When small numbers of 0.22 μm beads not coated with BSA were injected, some beads attached to cilia and, thus, the movement of several cilia in the wall of the ventricle could be visualised (Additional file 3: movie 3). In the boundary region between the third ventricle and the cerebral aqueduct, the beads moved along the walls of the ventricle toward the cerebral aqueduct (n = 21, Figure 3c, Additional file 4: movie 4). In the lateral ventricles, which lie anterior to the third ventricle, the CSF flow moved along the outer wall of each ventricle and then to the inner side, creating a semicircle (n = 19, Figure 3d, Additional file 5: movie 5). In the cerebral aqueduct, the beads moved rostrally in the dorsal region and caudally in the ventral region (n = 22, Figures 3e-h, Additional file 6: movie 6 and Additional file 7: movie 7). In the fourth cerebral ventricle, the pattern of bead flow in the dorsal region was quite different from that in the ventral region (n = 27, Figures 3i-j, Additional file 8: movie 8 and Additional file 9: movie 9). The beads moved from ventral to dorsal along the walls of the fourth ventricle and then moved proximally to the midline (Figure 3i, Additional file 8: movie 8). Near the floor of the ventricle, the beads moved from the midline towards the distal sides of the ventricle (Figure 3j, Additional file 9: movie 9). Almost all of the larvae survived for more than two days after the injections.

**Table 1:** Quantitation of the initial CSF flow pattern

Entrance of the beads from the cerebral aqueduct into the third cerebral ventricle			
**Enter from the left**	**Enter from the right**	**No difference**	**Others**

41%	20%	34%	5%

n = 190/467	n = 95/467	n = 160/467	n = 22/467

Pattern of bead movement from the cerebral aqueduct into the third cerebral ventricle.

**Table 2:** Quantitation of the continuous CSF flow pattern

Direction of the CSF flow pattern within the third cerebral ventricle				
**Clockwise**	**Counter clockwise**	**Symmetry**	**No-pattern**	**Others**

41%	13%	22%	14%	10%

n = 198/479	n = 62/479	n = 105/479	n = 68/479	n = 46/479

Pattern of the bead movements within the third ventricle a few minutes after the injection when the beads are uniformly distributed within the ventricles

To examine whether the experimental treatments affected the endogenous pattern of CSF flow, the injection position, injection volume, and size of the beads was varied. First, 2.3 nL of 0.96 μm beads were injected into a lateral cerebral ventricle at stages 47-48. Although the beads have a different diameter, the CSF flow pattern was similar to that of the 3.1 μm or 5.8 μm beads injected into the fourth cerebral ventricle (n = 9). The beads arrived at the fourth cerebral ventricle within a few minutes after the injection and then came back into the third cerebral ventricle by passing through the cerebral aqueduct. After the injection of 23 nL of 5.8 μm beads into one of the lateral ventricles at stage 46, the CSF flow was similar to that seen after standard injections into the fourth cerebral ventricle (n = 3).

To determine whether the beads themselves interfere with the normal pattern of CSF flow, beads of two different diameters were injected. First, 23 nL of blue 5.8 μm beads were injected into the fourth cerebral ventricle at stage 46. After 30 min, 13.8 nL of red 2.9 μm beads were injected into the larva at the same position as the first injection. The pattern of CSF flow did not change after the serial injections; the flow was the same as in single injections (n = 5). Taken together, these results suggest that the injection position, injection volume, and bead size did not affect the endogenous CSF flow pattern. Therefore, it can be considered that the described experiments reflect the endogenous CSF flow pattern in *Xenopus *larvae.

### Examination of stage-dependent changes in CSF flow pattern

**Figure 4:**
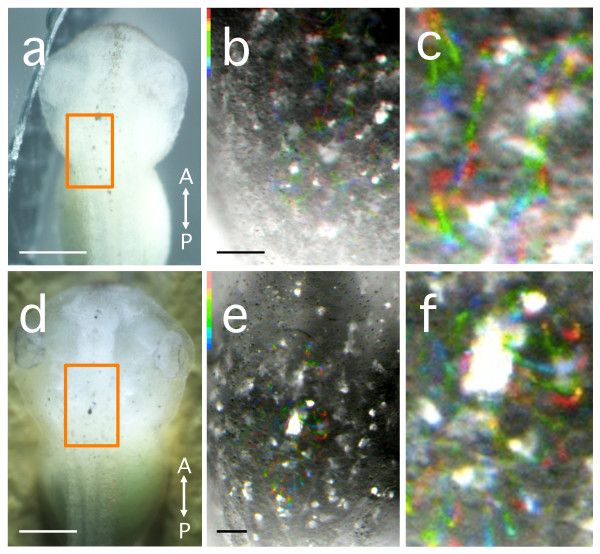
**Visualisation of bead movements injected into the fourth ventricle within the fourth ventricle in stage 39 (a-c) and stage 42 (d-f) anaesthetised non-feeding embryos**. Anterior is to the top. **(a) **Bright-field image of a stage 39 embryo (dorsal view). Orange box indicates the area of recording in (b). **(b) **Trajectories of the beads (diameter = 5.8 μm) within the fourth ventricle are shown in a separate embryo (dorsal view). The trajectories show the existence of some flow, but a discrete pattern was not apparent. **(c) **Magnified view of (b). Note the non-directional bead movements. **(d) **Bright field image of a stage 42 embryo (dorsal view). **(e) **Trajectories of the beads (diameter = 5.8 μm) within the fourth ventricle in the same embryo (dorsal view). Note that a radial CSF flow, from the centre of the ventricle toward the wall, is generated at stage 42. **(f) **Magnified view of (e). See Additional file 10: movie 10. Duration of recording; (b) 2.7 sec; (e) 0.9 sec. Scale bars, 0.5 mm (a, d) and 0.1 mm (b, e).

The developmental stage-dependence of the CSF flow pattern was examined to detect the stage at which CSF flow is produced. Beads (diameter = 5.8 μm) were injected into the fourth cerebral ventricle, using volumes of 2.3, 4.6, or 9.2 nL per embryo/larva at stage 38-39, stage 42-43, or stage 46, respectively. Stage 38-39 tailbud embryos are the earliest stage in which the cerebral ventricle is fluid-filled (Figure 2a), and CSF flow was observed in the fourth cerebral ventricle in these embryos (n = 9). However, it was not possible to determine a specific pattern of CSF flow at this stage (Figure 4a-c).

In stage 42-43 embryos, CSF flow could not be detected by injection of red beads (diameter = 0.96 μm or 2.9 μm), since the cranial epidermis is rather opaque at this stage. By injecting blue beads (diameter = 5.8 μm), CSF flow in the fourth ventricle could be observed, and the pattern of CSF flow was radial, originating from the centre of the ventricle (n = 16; Figures 4d-f, Additional file 10: movie 10). The cerebral aqueduct is not yet open at this stage (Figure 2b), and, thus, the injected beads could not enter the third cerebral ventricle via the cerebral aqueduct.

Injections into stage 46 larvae, in which bilateral expansion of the cerebral ventricle is half that of stage 47-49 larvae, revealed a CSF flow pattern very similar to that presented in Figure 3, although the distinct left-right individual differences in the flow into the third ventricle were absent (n = 21). The dorso-ventral difference in the CSF flow in the cerebral aqueduct has also been established in stage 46 larvae (n = 5). The CSF flow pattern in the third cerebral ventricle was bilateral with beads circulating anterior to posterior, which may be due to the narrow ventricular space. The flow around the choroid plexus of the third cerebral ventricle was the fastest observed, similar to the stage 47-49 pattern. After stage 50-51, the transparency of the dorsal side of the larvae is gradually lost, due to the thickening of the dorsal epidermis and cartilaginous skull, making it difficult to observe the CSF flow pattern. Additionally, inserting the glass capillary through the dorsal tissues became difficult (n = 13).

### Estimation of the volume of the larval ventricles

**Figure 5:**
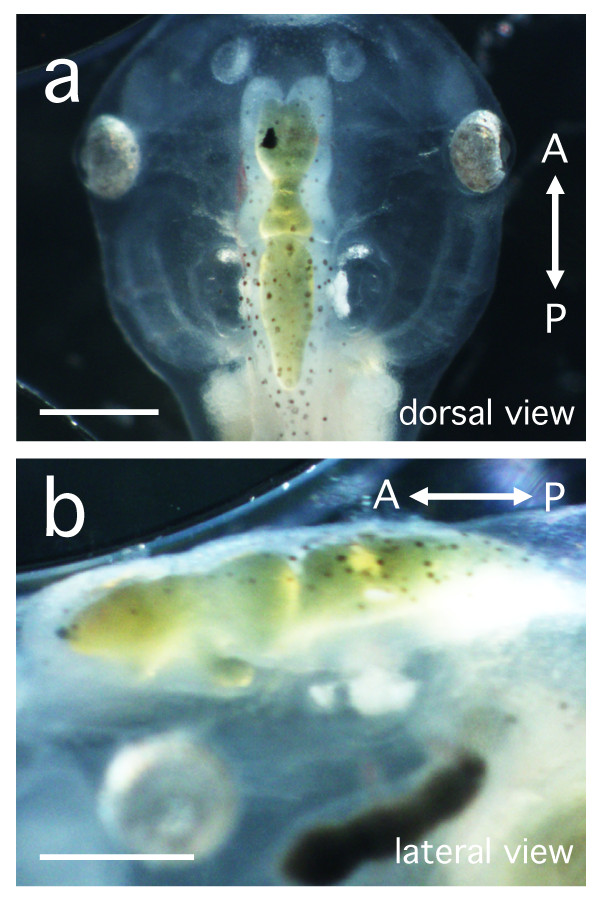
**Injection of oil into the *Xenopus *larval ventricles to estimate their volumes**. A living stage 46 larva after injecting 87 nL of sesame oil into the brain ventricles. The yellow sesame oil fully fills the cerebral ventricle of the larva, as observed by the morphology of the ventricles (**a**, dorsal view; **b**, lateral view). Scale bars, 0.5 mm.

To estimate the total volume of the larval brain ventricles, we injected sesame oil into the ventricle. As the injection volume increased, the cerebral ventricle gradually became filled with the injected oil in one large droplet, filling the complex ventricular shape without internal damage (n = 17; Figure 5). In stage 46 larvae, 19 injections of 4.6 nL (i. e. 87.4 nL) were conducted without leaking. After 20 such injections, the oil was observed leaking out of the injection site in five out of eight larvae when the injection capillary was removed. These results indicate that the volume of the cerebral ventricle in stage 46 larvae is between 87 and 92 nL. In stage 50 larvae, the volume of the cerebral ventricle was estimated to be 3.2-3.9 × 10^2 ^nL by the same method (n = 10). When the oil-injected larvae were viewed laterally, the oil could be seen penetrating into the hollow of the infundibulum (Figure 5b). Based on these volume estimations, an injection of 4.6 nL of beads was equivalent to a volume of 5.3% or 1.4% of the total volume of the ventricles at stages 46 and 50, respectively.

## Discussion

Since Qdot particles, as used in our studies, have high fluorescence intensity, low cytotoxicity, and are resistant to bleaching, they have become popular as fluorescent markers in cell biology experiments [18-20]. In the current study, the fluorescent images produced following Qdot565 injection revealed dynamic changes in the morphology of the cerebral ventricle with age (Figure 2). These changes may reflect cell growth in relation to the segmentation of developing brain tissue, the physiological response of the neuroblasts migrating in the subventricular zone to intracranial pressure, and the mechanics of the developing cranial tissue constricting the cerebral vesicle [21-25].

We developed a new method to observe the orchestrated flow of CSF by combining the microinjection of beads with microscopic observations. This technique enabled us to observe CSF flow in living specimens. Previously, in mouse embryos and newborn mice, CSF fluid dynamics based on ciliated cell activity have been examined in detail, but the observations were conducted using dissected brain tissue cut in half to expose the inner surface of the brain ventricles [16,26]. This approach was used because the tissues lack transparency needed for intact viewing. The current study of CSF flow in albino *Xenopus *larvae allowed us to obtain complementary knowledge from a living specimen with minimal injury.

In the sagittally-dissected neonatal mouse, CSF in the lateral ventricles flows from the caudal to rostral direction, and results in migration of neuroblasts toward the olfactory bulb. This neuronal migration, called the rostral migratory stream (RMS), was first linked to CSF flow by Sawamoto *et al. *[26] in rodents, and RMS has also been found in primates and humans [27,28]. In contrast, when viewed from the dorsal side, *Xenopus *larval lateral ventricles exhibited a semicircular flow of CSF, from the distal to medial walls (Figure 3d). In adult human brain, CSF is secreted by the bilateral choroid plexuses, making a laminar flow through the lateral ventricles and exits through the foramina of Monro into the third ventricle. The CSF then flows into the fourth ventricle and leaves through the foramen of Magendie and the foramina of Luschka, flowing into the subarachnoid space before being absorbed into the superior sagittal sinus [29]. The most prominent difference in CSF flow between *Xenopus *larvae and mammals is that the former exhibits rapid circulation within the ventricles and "counter flow" from the fourth ventricle to the third ventricle through the dorsal region of the cerebral aqueduct. This finding suggests that we should clarify the rules of CSF flow patterning in vertebrates, with special awareness to discriminate between what is conserved and what is not conserved across vertebrate species.

Because *Xenopus laevis *is a well-studied model organism, a number of homologous genes involved in the planar cell polarity (PCP) pathway, intraflagellar transport (IFT), and ciliogenesis have been identified [30-32]. Thus, the molecular mechanisms of CSF flow patterning can be examined and compared to the mechanisms elucidated in mammals. The present study revealed the basic CSF flow pattern in *Xenopus *larvae and how it changes with developmental stage. Consequently, future studies can examine the responses of the global CSF flow patterning to changes in pH, salinity, CSF components, or knocking down the components needed for ciliogenesis in ependymal cells.

In the later stages of mouse embryos and in newborn mice, normal CSF flow requires two, mutually-independent polarities in the ependymal cells [33,34]. These are called "rotational polarity" (polarity of the tilt direction of the rotational axis of each cilium) and "translational polarity" (polarity of the position of cilia formation on the apical surface of the ciliary epithelium) [35]. These two polarities are established by different mechanisms. Radial glial cells are the precursors of ependymal cells, and their primary cilia have already established a translational polarity within several days of birth [33]. In neonatal mice, PCP in ependymal cells guides the direction of CSF flow [36], and, in turn, the initial CSF flow caused by the secretion and absorption of the fluid influences the directionality of ciliary beating by the ependymal cells [37].

In contrast, *Xenopus *brain ventricles after larval stage 38, generate closed circular movements of CSF, which, by stage 46-53, become highly orchestrated and stable (see Figures 2 and 3, Additional file 1: movie 1, Additional file 2: movie 2, Additional file 4: movie 4, Additional file 5: movie 5, Additional file 6: movie 6, Additional file 7: movie 7, Additional file 8: movie 8, and Additional file 9: movie 9). The directionality of CSF flow in late larvae is invariant. The circulation of *Xenopus *larval CSF may mediate the establishment of polarity of the ciliary ependymal cells by mechanisms that differ from those in mice. In young *Xenopus *larvae, the initial patterning of the CSF flow may be regulated by the ciliated choroid plexus of the third and fourth ventricles before active ciliogenesis is initiated in the ependymal cells [38].

Kramer-Zucker *et al. *[39] traced the diffusion of CSF in newly hatched zebrafish, but the detailed pattern of zebrafish CSF flow has not yet been elucidated due to the relative opacity of the fry. Recently Kishimoto *et al. *[40] reported in adult zebrafish that a cluster of neuronal precursors generated in the telencephalic ventricular zone migrates into the olfactory bulb via a pathway equivalent to the mammalian rostral migratory stream (RMS). That study suggests that ventricular walls of adult vertebrates are conserved niches of adult neurogenesis. *Xenopus *larval brain will provide a good model system to investigate the correlation between migration of the olfactory neuroblasts and CSF flow in the lateral ventricles. In *Xenopus *larvae, the olfactory bulbs are near the lateral ventricles, the olfactory nerves and olfactory pits are semi-transparent, and, importantly, they are positioned linearly, facilitating quantitative observations when viewed from the dorsal side.

In Celsr 2/3 (PCP cadherins) double-knockout mice, ependymal ciliogenesis was perturbed and this caused CSF flow defects leading to lethal hydrocephalus [41]. In our future experiments, after morpholino knockdown of ciliary components or of essential components in the ependymal PCP pathway, changes in the CSF flow pattern will be examined and the migration pattern of neuroblasts will be determined. Such experiments will clarify the significance of CSF flow patterns for the developing brain.

## Conclusions

Real-time observations of the relationship between CSF flow and the development of the CNS have been made *in vivo *in *Xenopus *larvae after the injection of nanocrystals or polystyrene beads into the fourth ventricle. CSF flow showed a consistent pattern of semicircular movement in the lateral ventricles, and a dorso-ventral antero-posterior pattern in the cerebral aqueduct and fourth ventricle. In the third ventricle there were individual differences in left-right asymmetrical flow. This new model system will be useful for future analyses of the pathology of diseases related to CSF flow, such as hydrocephalus or intracranial hypotension syndrome.

## Abbreviations

CSF: cerebrospinal fluid; cp: choroid plexus; ca: cerebral aqueduct (aqueduct of Sylvius); lv: lateral ventricle; BSA: bovine serum albumin.

## Competing interests

The authors declare that they have no competing interests.

## Authors' contributions

KM and RT carried out the microinjection experiments and video processing with Image-J and its plug-in. TA and SI were involved in the writing of the Results and Discussion sections, as well as critical reading of the manuscript. All authors have read and approved the final version of the manuscript.

## Supplementary Material

Additional file 1 **Asymmetric CSF flow entering into the third ventricle of a stage 47 larva**. *Xenopus *larval CSF flow is detected by the movement of polystyrene beads injected into the fourth cerebral ventricle. Beads entering the third ventricle from the cerebral aqueduct (aqueduct of Sylvius) show distinct left-handed CSF flow. This movie corresponds to Figure 3a. Click here for file

Additional file 2 **Continuous CSF flow in the third ventricle of a stage 48 larva**. In the third ventricle, in many cases, the fluid flow shows left-right asymmetrical flow, and it is faster near the choroid plexus. This movie corresponds to Figure 3b. Click here for file

Additional file 3 **Ciliary movement at the inner wall of the third ventricle of a stage 47 larva**. This movie shows the motility of the cilia to which a few to several 0.22 μm beads adhered. These cilia are distributed in the left rear wall of the third ventricle, and protrude into the third ventricle. Note the circular rotation of the bead-coated cilia, which might play a role in generating the CSF flow pattern. Click here for file

Additional file 4 **CSF flow around the rear opening of the third ventricle of a stage 48 larva**. In the boundary region between the third ventricle and the cerebral aqueduct (aqueduct of Sylvius), the beads enter the cerebral aqueduct along the wall of the third ventricle. This movie corresponds to Figure 3c. Click here for file

Additional file 5 **CSF flow in the lateral ventricles of a stage 48 larva**. In both of the lateral ventricles, the fluid flow circulates from the outer side to the inner side and then recurrently returns into the third ventricle. This movie corresponds to Figure 3d. Click here for file

Additional file 6 **CSF flow in the *dorsal *region of the cerebral aqueduct of a stage 48 larva**. In the dorsal region of the cerebral aqueduct, the flow moves towards the anterior. This movie corresponds to Figure 3e. Click here for file

Additional file 7 **CSF flow in the *ventral *region of the cerebral aqueduct of a stage 48 larva**. In the ventral region of the cerebral aqueduct, the flow moves towards the posterior, in contrast to Additional file 6: movie 6. This movie corresponds to Figure 3f. Click here for file

Additional file 8 **CSF flow in the *upper *region of the fourth ventricle of a stage 48 larva**. In the upper region of the fourth ventricle, the flow concentrates toward the centre of the ventricle with anterior shifting. This movie corresponds to Figure 3i. Click here for file

Additional file 9 **CSF flow in the *lower *region of the fourth ventricle of a stage 48 larva**. In the lower region of the fourth ventricle, the flow disperses to the periphery with posterior shifting, in contrast to Additional file 8: movie 8. This movie corresponds to Figure 3j. Click here for file

Additional file 10 **CSF flow within the fourth ventricle of a stage 42 embryo**. In contrast to the CSF flow in stage 39 embryo (Figure 4a-c), a radial flow pattern could be observed in the fourth ventricle. This movie corresponds to Figure 4d-f. Click here for file
